# Manipulating Individual Topological Solitons and Bisolitons in an Electronic System

**DOI:** 10.1002/adma.202510318

**Published:** 2025-09-25

**Authors:** Taehwan Im, Jae Whan Park, Han Woong Yeom

**Affiliations:** ^1^ Center for Artificial Low Dimensional Electronic Systems Institute for Basic Science (IBS) Pohang 37673 Republic of Korea; ^2^ Department of Physics Pohang University of Science and Technology Pohang 37673 Republic of Korea

**Keywords:** charge density waves (CDW), multi‐valued information processing, soliton manipulation, scanning tunneling microscopy (STM), *Z*_4_ topology, topological solitons

## Abstract

While localized topological modes in quantum materials, such as topological solitons and Majorana fermions, promise loss‐less delivery of classical and quantum information, manipulating individual topological modes has been highly challenging. The manipulation of Z_4_ topological solitons is reported in a 1D charge density wave (CDW) insulator of indium atomic wires on a silicon surface. Using the current injection from a scanning tunneling microscopy tip, individual defects‐pinned solitons can be created, translated, and annihilated. Moreover, this method is applied to induce bi‐soliton processes such as transforming a soliton into a different soliton, fissioning one into two solitons, and fusing and dissociating two solitons, where soliton topological charges are manipulated. The theoretical calculations attribute the mechanism of manipulation to local destabilization of the CDW structure by hole doping. This work demonstrates addressable control over most of the possible operations between individual and paired *Z*_4_ solitons and secures a way to investigating dynamics of quantum solitons and to processing topologically‐protected multi‐valued information in electronic systems.

## Graphical abstract

While localized topological modes in quantum materials promise loss‐less delivery of classical and quantum information, manipulating them individually has been challenging. This work succeeded in manipulating topological solitons formed along atomic wires on silicon surfaces with high reproducibility in various ways, including translating, transforming into a different type of solitons, fissioning into two solitons, and fusing and dissociating two solitons.

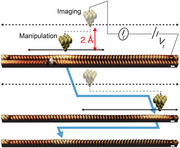

## 1 Introduction

With the rapid recent growth of computing needs, the ability to process vast volume of information energy‐efficiently has become increasingly demanding.^[^ ^1^, ^2^, ^3^, ^4^ ^]^ While various research efforts have been made to handle much larger volume of information with much lower energy consumption,^[^ ^5^, ^6^, ^7^, ^8^, ^9^, ^10^, ^11^ ^]^ dissipation‐less carriers of information in electronic systems may have fundamental impact in future device technology. Such carriers can in principle be realized by topological protection to make them immune to scatterings as in the cases of localized topological modes such as topological solitons in topological insulators and Majorana fermions in topological superconductors.^[^ ^12^, ^13^ ^]^ Not only classical information, but also quantum information may be processed with solitons^[^ ^14^, ^15^ ^]^ and Majorana fermions.^[^ ^16^, ^17^, ^18^ ^]^ While recent investigations showed that addressing Majorana fermions is extremely challenging,^[^ ^19^ ^]^ individual topological solitons were unambiguously identified by scanning tunneling microscopy in *Z*_3_ and *Z*_4_ 1D topological insulators.^[^ ^12^, ^20^, ^21^ ^]^ In these systems, three or four different types of solitons exist with different electronic states and charges indicating the possibility of handling multi‐valued information beyond a binary system. Namely, topological solitons have the potential to process large volume of classical or quantum^[^ ^14^, ^15^ ^]^ information in a dissipation‐less way.

However, manipulation of individual solitons or Majorana fermions has not been demonstrated^[^ ^12^, ^20^, ^22^ ^]^ to prevent further development of topologically protected carriers in electronic systems.^[^ ^15^, ^23^ ^]^ This is contrasted with the classical soliton technologies established well in optical systems for long‐distance communication. These technologies are based on the creation and the transportation of solitons and the creation/dissociation of their bound states using electric fields of light.^[^ ^24^, ^25^, ^26^ ^]^ Similar technologies are under active development for solitons in spin systems, skyrmions,^[^ ^27^, ^28^, ^29^, ^30^, ^31^ ^]^ and limited controllability has been reported for matter wave solitons in cold atom chains.^[^ ^32^, ^33^ ^]^ We focus our attention on *Z*_4_ topological solitons discovered in indium atomic wires self‐assembled on a Si(111) surface.^[^ ^12^, ^20^ ^]^ This system is one of the first cases of successful microscopic observation of individual mobile solitons in electronic systems, allowing the access to individual topologically‐protected carriers in an electronic system. However, the artificial control over *Z*_4_ solitons has not been demonstrated and only very limited control over *Z*_3_ solitons in a similar atomic wire system, that is, the pair creation of solitons, was reported recently.^[^ ^21^ ^]^

In the present work, we demonstrate most of the essential manipulation steps over individual solitons and pairs of solitons^[^ ^34^ ^]^ by local hole current injection, including translation between pinning sites, pair creating and annihilation, fission, fusion, and transformation. The underlying mechanisms of the manipulation were revealed through electronic structure calculations. This work manifests that topological solitons in electronic systems are fully manipulatable and addressable systems toward future device technologies, which can be dubbed as solitonics, and provides a platform to investigate microscopic interactions of quantum solitons in solids.

## 2 Results and Discussion

### 2.1 Topological Solitons in Indium Atomic Wires

**Figure 1:**
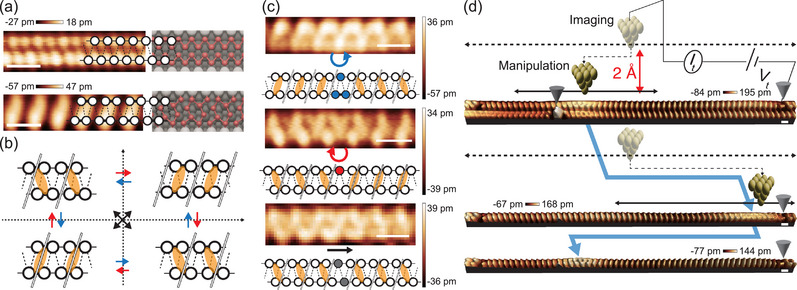
*Z*_4_ solitons along the indium CDW wire and the STM manipulation over a soliton. a) STM images (tunneling bias *V*_*t*_ = –500 mV and current *I*_*t*_ = 0.1 nA) of an indium wire on Si(111) in the 4×1 metallic phase (top panel) and the 4×2 CDW phase (bottom), together with the corresponding double‐chain SSH model overlapped with the atomic structures of the indium chain on the Si(111) surface. In the atomic structures, red and white spheres represent indium and silicon atoms, respectively. The dimerization is indicated by thick solid lines and the CDW maxima by orange ellipses. b) SSH model schematics of the four degenerate CDW states with different dimerization configurations within a CDW unitcell, which are topologically distinct and connected by three distinct topological domain walls indicated by blue, red, and black arrows. (c) STM images (*V*_*t*_ = –500 mV, *I*_*t*_ = 100 pA) and SSH model schematics of the domain walls, which correspond to RC, LC, and NC solitons (from top to bottom). The full description of the ground states, the soliton domain walls, the topological structure, and the atomic structures are detailed in Figures S1, S2, S3, S6, and S7 (Supporting Information). d) Sequential STM images (‐500 mV, 0.1 nA) of two indium wires with one RC soliton and two pinning defects (indicated by grey cones). The bright defect located in the bottom wire pins the soliton in the top wire, which contains a dark defect at the right end that pins the manipulated soliton. The vertical and lateral motions of the STM tip (illustrated schematically) for imaging and manipulation (–500 mV and 0.9 nA) are illustrated by dashed and solid arrows. The STM tips are schematically shown. The location of the RC soliton is tracked by blue thick lines. After the first depinning, we imaged only one wire to track the moving soliton quickly as shown in the middle and the bottom images. The translucent and solid yellow spheres forming a tetrahedron in the top two wire diagrams represent the imaging and manipulation tips, respectively. All STM images were captured at 78 K and the scale bars represent 1 nm.

The deposition of a monolayer of indium atoms on a clean Si(111) 7×7 surface results in the self‐assembly of metallic wires consisting of double zigzag chains of indium atoms in a 4×1 unitcell.^[^ ^20^, ^35^, ^36^ ^]^ Below 125 K, indium chains undergo Peierls distortion into an 4×2 tilted‐hexagon structure as shown in **Figure** **1**a.^[^ ^20^, ^35^, ^36^ ^]^ The distortion introduces an alternation of short and long In‐In bonds along the outer In rows, which can be mapped into a double‐chain Su–Schrieffer–Heeger (SSH) Hamiltonian (Note SI and Figure S1, Supporting Information).^[^ ^20^ ^]^ The double chain structure yields not two but four degenerate CDW structures of a *Z*_4_ topology (Figure 1b), which naturally leads to three different soliton domain walls called right‐chiral (RC), left‐chiral (LC), and nonchiral (NC) solitons (Figure 1b,c).^[^ ^20^ ^]^ These solitons are endowed with distinct atomic structures (Figure 1c), in‐gap electronic states (Figures S1 and S2, Supporting Information), and topological charges (Figure S3, Supporting Information). It has been found that the solitons are highly mobile, but the chiral (RC and LC) solitons are easily trapped by extrinsic defects within the same wire or in a neighboring wire (see Figure 1d).^[^ ^20^, ^37^, ^38^ ^]^

### 2.2 Manipulation of Individual Solitons via Local Hole Current

**Figure 2:**
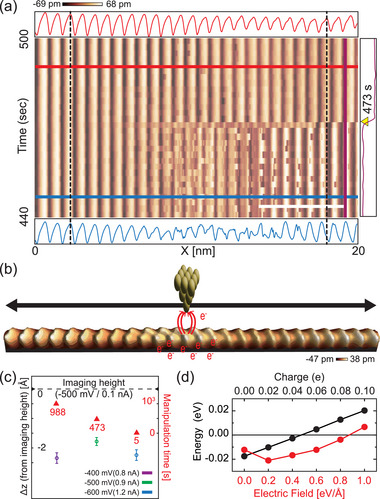
Mechanism of soliton translation. a) Time‐sequence STM line scan along the center of the wire in (b) at *V*_*t*_ = –500 mV, *I*_*t*_ = 0.9 nA. Each line profile was acquired in 1.0 s with an interval of 2.0 s using bidirectional scans (only forward‐scan data are shown for simplicity). The depinning event (yellow arrowhead) occurs at 473 s. Blue (before) and red (after) profiles indicate the presence (absence) of a soliton before (after) the depinning event occuring at 473 s, when the CDW maxima changes to minima (shown in the purple line) due to the CDW phase shift introduced by the soliton motion. Fluctuations of the CDW state (the noisy line profiles) are observed before depinning. All STM images were taken at 78 K; scale bars, 5 nm. b) Schematics of the tunneling (hole doping) process with a STM image (*V*_*t*_ = –500 mV, *I*_*t*_ = 0.1 nA) of an indium wire containing a RC soliton. c) The tip height and the time measured before the soliton depinning. (d) Change of the total energy of the 4×2 CDW structure compared to the 4×1 structure calculated by DFT under the hole doping (red circles) and the out‐of‐plane electric field (black circles).

The manipulation of a single soliton pinned to a defect in the neighboring wire is schematically illustrated in Figure 1d. We scanned over a wire segment (20 nm in length) containing a trapped RC soliton with an STM tip at 500 mV bias and systematically increased (decreased) tunneling current (tip‐sample distance). When the tunneling current reached 0.9 nA (the tip lowered by 0.2 nm), the soliton was depinned to move to the right end of the wire, where it was imaged at a lower current to be trapped to an intrawire defect. This process can be reversed to move the soliton back to the original pinning site (Figure 1d; Figure S4, Supporting Information). The real‐time STM line scans under the high tunneling current (**Figure** **2**a) show how the soliton is de‐pinned in more detail. One can notice that the CDW state of the indium chain fluctuates (its line scan becoming noisy) until the soliton is depinned to move to the right. This fluctuation occurs before every manipulation events (Figure S5, Supporting Information) and indicates that the CDW state is destabilized in this condition. We found that the depinning occurs only at a negative bias, electrons (holes) tunneling into the tip (the samples), of a magnitude higher than 300 mV over a current around 1.0 nA (Figure 2; Figure S5, Supporting Information). The critical tunneling current and Δ*z* (relative to the imaging height) shows little variation across different bias voltages; however, the total scan time required for depinning is significantly reduced at –600 mV, as shown in Figure 2c. This indicates that the energy barrier of depinning is reduced at a high bias as analyzed below. That is, the tunneling‐induced manipulation requires the hole injection into the sample and a sufficiently high bias.

### 2.3 Energetics for Soliton Dynamics

Based on the previous findings that a silicon surface can experience transient doping under tunneling current and that the CDW state is sensitive to the doping level,^[^ ^39^, ^40^, ^41^, ^42^ ^]^ we postulate that the fluctuation and the subsequent soliton depinning is induced by the hole doping due to the tunneling electron flow from the sample to the tip. This scenario is supported by energetic considerations through density functional theory (DFT) calculations. Figure 2d shows the energy gain of the CDW formation as a function of vertical electric field strength and doped hole concentration (represented by the blue and black lines, respectively). The CDW state is destabilized with hole doping as expected^[^ ^39^, ^40^, ^42^ ^]^ even with a small amount of positive charge (∼0.02 e per a 4×2 cell). On the other hand, small electric field enhances the stability of the CDW state while a bigger field than 0.6 eV/Å has an opposite effect (Figure 2d). The band manifold shifts upward almost rigidly with the top of the indium‐driven filled states brought into the empty state upon hole doping while the vertical electric field induces charge transfer from the Si substrate to the indium chain to move the band in an opposite direction (Figure S6, Supporting Information). These calculations explain the forbidden and the enhanced probability of the manipulation at low and high bias, respectively, and the competing effects of the electric field and tunneling current on the sample (as shown in Figures S8 and S9, Supporting Information). The destabilization of the CDW means the reduction of the CDW amplitude, which also reduces the energy barrier for the CDW state to relax into an undistorted state through its phase and amplitude fluctuations (Figure S6, Supporting Information). Such destabilization, thus, would facilitate the propagation or deformation of a topological soliton in which the CDW has a phase shift and zero amplitude.

**Figure 3:**
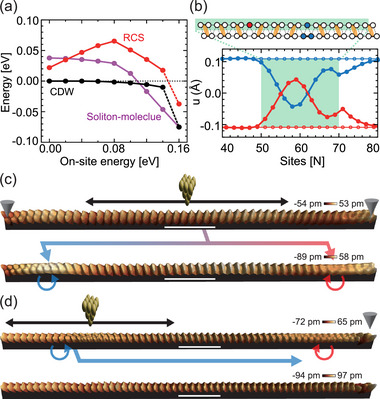
Pair creation and annhilation of solitons. a) Relative total energies of a bare CDW chain, a chain with a RC soliton, and one with a soliton molecule, which are calculated by a 200‐site tight‐binding model as function of the local on‐site energy (over 21 sites) around the soliton. Both a RC soliton and a soliton molecule become stable at a sufficiently large on‐site energy increase, while a RC soliton becomes unstable at a smaller on‐site energy. b) Lattice distortion amplitude *u* for each site of a 21‐site chain under the SSH model. The red (blue) symbols represent atoms in the odd(even)‐numbered sites displaced in the negative (positive) direction from the undistorted 4×1 configuration, forming alternating dimerized pairs in the ground‐state CDW phase. Open circles denote the ground‐state CDW configuration without on‐site energy perturbation, while filled circles show the dimerization pattern with an on‐site energy of 0.16 eV applied for 21 sites (denoted by the green area) (Figure S11, Supporting Information). The perturbed dimerization patterns with two zero distortion points indicates the formation of two solitons as depicted in the schematic model in the top pannel. c) STM images (*V*_*t*_ = ‐500 mV, *I*_*t*_ = 100 pA) of a bare CDW state with intrawire defects on both sides (indicated by arrow heads) before (top) and after (bottom) of the manipulation. A RC (blue arrow) and a LC (red arrow) soliton was created and trapped by the defects. The CDW phase map (phase zero to π in a red‐to‐blue color scale) indicates the existence of solitons quantitatively. d) Similar STM images of a RC and a LC soliton before (top) and after (bottom) the manipulation. The two solitons are annihilated after the scanning at a higher current (–500 mV, 900 pA) over the RC soliton on the left. All STM images were captured at 78 K and the white scale bars represent 5 nm.

Since DFT calculations for huge unit cells containing solitons with extra charge or electric field are practically not feasible, the relaxation of a soliton under local hole doping was investigated by tight‐binding calculations (Figures S10 and S11, Supporting Information). **Figure** **3**a presents the total energy of the double‐chain SSH model with and without a soliton as a function of the on‐site energy. Once the the Fermi level is fixed, the band filling changes according to the on‐site energy, which corresponds to doping. To model the local perturbation by the tunneling current, we adjust the on‐site energies of 21 sites (∼8 nm) out of 200 total sites in each chain.^[^ ^43^ ^]^ This choice is reasonable when one considers the STM condition in which the tip–sample distance is 1–2 nanometers and the typical tip radius is 20–30 nm^[^ ^43^, ^44^ ^]^ (see for the details in Figure S12, Supporting Information). The system with a preformed RC‐soliton has an excitation energy of 0.02 eV initially but it increases up to 0.08 eV (red line in Figure 3a) as the on‐site energy increases locally. Thus, the RC‐soliton becomes unstable to favor propagation out of the perturbed region. This calculation aligns qualitatively with our observations and the above DFT results. The calculation reveals a contrasting trend for a LC soliton, becoming more stable under the on‐site‐energy increase (blue dots in Figure S10 and S13, Supporting Information) and, indeed, a LC soliton was not easily manipulated with the present condition (Figure S13, Supporting Information). These calculations and their agreement with the observations tell consistently that increasing doping level by the increased current reduces the energy barrier for the soliton translation. On the other hand, the electric field effect (±*V*) can be expressed in terms of the chemical potential as µ = ±*eV*.^[^ ^43^ ^]^ To isolate the pure electric field effect, one must maintain an overall half‐filled condition so that no doping occurs. The corresponding calculation, however, shows that the electric field has only a marginal effect (see Figure S12, Supporting Information). Using the total current injection times until the depinning events at –400 mV (0.8 nA) and –500 mV (0.9 nA), we can estimate the energy barrier through the Arrhenius formula, *D* = *D*_0_exp (−*E*_*b*_/*k*_*B*_ *T*). Under the comparable prefactors *D*_0_, the ratio of two different event rates depends only on the difference in barrier heights Δ*E*_*b*_ and can be expressed as ∼exp (−Δ*E*_*b*_/*k*_*B*_ *T*). In the present experiment, the event‐rate ratio is 473/988 between –400 and –500 mV cases and the Δ*E*_*b*_ is estimated as about 5 meV at 78 K. This value matches reasonably the energy scale calculated in DFT under hole doping (Figure S6, Supporting Information). It is also worth noting that STM‐induced local heating has been reported to be only in the order of few kelvins even under high tunneling currents.^[^ ^45^, ^46^ ^]^ This effect thus should be much small than the hole doping effect.

### 2.4 Creation, Annihilation, and Transformation of Solitons

In principle, providing a sufficient energy locally would create a pair of a soliton and an anti‐soliton. Indeed, under the same hole‐current injection by the STM tip as the depinning, we can easily create a RC‐LC soliton pair from a pristine CDW wire as shown in Figure 3c. Moreover, one can inject tunneling current into the wire containing a RC and a LC soliton nearby to move the RC soliton and, subsequently, annihilate two solitons in pairs (Figure 3d). The pair‐creation and pair‐annihilation are hallmarks of the *Z*_2_ topological nature of a soliton. In the algebra of topological charges of the *Z*_4_ solitons,^[^ ^12^ ^]^ RC and LC solitons are the proper pair to be pair‐created and ‐annihilated while the pairing with a NC soliton converts a RC (LC) soliton into a LC (RC) soliton.^[^ ^12^ ^]^ The latter was indirectly suggested by observing the time‐dependent switching between RC and LC solitons in the previous STM experiment.^[^ ^12^ ^]^ One can theoretically examine the effect of local hole doping on a pristine CDW state using the above tight binding model. The system retains its CDW order with minor distortions (<0.03 Å) up to an on‐site energy of 0.14 eV (Figure S11, Supporting Information) but a sharp drop in total energy is observed beyond 0.16 eV (black dashed line in Figure 3a), which is accompanied by a change in the dimerization pattern of the indium chain as shown in Figure 3b. In Figure 3b, the red and blue curves indicate the displacements of atoms in the CDW state at odd and even‐numbered sites, respectively. The open symbols represent the unperturbed dimerized ground state, whereas the filled symbols correspond to those perturbed by an on‐site energy of 0.16 eV, which indicates the formation of a pair of RC and LC solitons (the two zero‐displacement points).

**Figure 4:**
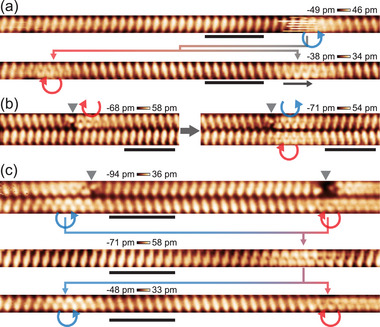
Fission and transformation of a soliton and fusion and dissociation of two solitons. a) Similar STM images of a RC soliton (top), which was manipulated to fission into a LC and a NC soliton (black arrow) (bottom). b) (Left) STM images (‐500 mV, 20 pA) of two indium wires where the upper wire contains a LC soliton trapped to an intrawire defect (black arrow head). We translate a LC soliton in the bottom wire from out of the image, which is captured by the defect‐solton composite of the top wire. In this event, the LC soliton in the top wire was converted to a RC soliton and the CDW state of the top‐right part of the wire was changed accordingly. This makes the CDW phase into a proper 8x2 order in both left and right parts of the soliton. c) Similar STM images of a RC and a LC soliton for two sequential manipulations. For the first manipulation scan (–600 mV, 1.2 nA) over the RC soliton, the two solitons form a soliton molecule^[^ ^34^ ^]^ and the second manipulation scan over the molecule dissociates it back to the two solitons. All STM images were captured at 78 K and the white scale bars represent 5 nm. For clarity, enlarged STM images are provided in Figure S14 (Supporting Information).

Since the pair creation and annihilation is only a part of the soliton operations within the *Z*_4_ soliton topology, we tested further manipulations of bi‐solitonic processes. We injected tunneling current into an RC soliton and found that it fissions into a pair of a LC and a NC soliton as shown in **Figure** **4**a. The process occurs whenever we apply the tunneling current into a fluctuating RC soliton, which is identified by the noisy STM image as shown in Figure 4a and Figure S15g (Supporting Information). This agrees well with the *Z*_4_ topological structure, mentioned above.^[^ ^12^ ^]^ The transformation of an RC (LC) soliton into a LC (RC) soliton can also artificially be induced; when a LC soliton is moved close to the other LC soliton trapped in the neighboring wire, the trapped soliton is converted to a RC soliton as shown in Figure 4b. This can be due to the substantial interwire interaction to favor the out‐of‐phase CDW, which requests the alternation of an RC and LC solitons in neighboring wires. The tunneling current injection into a wire with a RC and a LC soliton can also lead to the fusion into a soliton molecule, a bound state of two solitons observed recently,^[^ ^34^ ^]^ instead of the pair annihilation as shown in Figure 4c. The cases of the pair annihilation and the molecule formation can be distinguished by the defect configurations; when the LC soliton is pinned by an intrawire (interwire) defect the pair annihilation (the molecule formation) occurs. This is consistent with the recent observation that the soliton molecule is stabilized by the presence of defects in the neighboring wire, which can affect the interaction potential between two solitons.^[^ ^34^ ^]^ The soliton molecule can easily be dissociated at the same manipulation condition (Figure 4c). The transformation between RC and LC solitons and their fusion into a molecule is beyond the *Z*_4_ topology, indicating the existence of extra interactions between solitons. Such interactions need further investigation but they, in turn, provide wider manipulability over solitons.

We note that the above procedures, especially the basic manipulations such as the pinning‐depinning and the translation are highly controllable and reproducible. With the manipulation condition established, we can reproduce the same procedure more than ten times at the same location and at many different locations without a single failure. We also confirmed the reproducibility of all other types of manipulations on different locations (see Figure S15, Supporting Information, for details); we could reproduce without a failure if the same configurations of defects and soliton types are secured. The only manipulation involving probabilistic procedure is the LC‐RC soliton transformation (Figure 4b), where a moving LC soliton in a right direction should be created through the pair creation. Such high reproducibility manifests that most of these operations are governed robustly by the topology of the system. While a large part of the manipulations demonstrated here depends on the interaction with defects, the defect‐free manipulations are alo possible. For example, one can pin and depin a soliton at the terminating ends of a wire segment (Figure S15a, Supporting Information) and the pining site can be provided by a soliton in the neighboring wire (Figure S15a, Supporting Information). Moreover, the atomic structure of the pinning defect is well known^[^ ^47^, ^48^ ^]^ and its density can be widely controlled.^[^ ^49^ ^]^ The highly robust manipulations and the controllability over the defect density make the present soliton system a promissing platform to further investigate soliton‐soliton and soliton‐defect interactions and utilize them for devices.

## 3 Conclusion

Our work demonstrates a wide range of manipulability over individual and pairs of topological solitons using the *Z*_4_ solitonic system of self‐assembled indium atomic wires, which includes translation between pinning sites, pair‐creation/annihilation, fusion, fission, and transformation. This achievement represents the first such manipulations over localized topological modes in electronic systems to the best of our knowledge. The present method opens up an avenue toward fundamental studies on kinetics and dynamics of solitons and novel in‐gap quasiparticle excitations emerging from soliton interactions in electronic systems. On the other hand, the present material platform and methodology can lead to multi‐valued memory and logic operations based on highly‐mobile and topologically‐protected information carriers, which may be utilized in energy‐efficient processing of a vast volume of information. Realizing novel qubits with solitons can be envisioned in forthcoming studies based on the bisolitonic manipulations.

## 4 Experimental Section

The experiment was conducted in an ultra‐high vacuum system equipped with a commercial cryogenic STM system. The In/Si(111)‐(4×1) surface was prepared by depositing one monolayer of indium atoms onto a Si(111)7×7 surface kept at 600 K, which was cleaned by flash heatings at 1500 K.^[^ ^35^ ^]^ The sample was then cooled down to 78 K for STM measurements, which is well below the CDW transition temperature of 125 K.^[^ ^35^, ^50^ ^]^ STM images were obtained at a tunneling current of 100 pA, 900 pA and a sample bias of ± 500 mV. The STS data were measured using the lock‐in technique with a modulation voltage of 20 mV. DFT calculations were carried out using the Vienna Ab initio Simulation Package (VASP) within the local density approximation.^[^ ^51^, ^52^ ^]^ To model the Si(111) surface, we employed a periodic slab geometry with six silicon layers for the pristine (8 × 2) structure, separated by a vacuum of approximately 13 Å. The bottom of the slab was passivated with hydrogen atoms. Electronic wave functions were expanded using a plane‐wave basis set with a kinetic energy cutoff of 400 eV, and Brillouin‐zone integration was performed with a 3 × 11 × 1 k‐point grid for the 8 × 2 structure. All atoms, except the bottom two silicon layers, were relaxed until residual forces fell below 0.02 eV/Å. For the electric field calculations, a correction was applied to account for the contact potential difference caused by the asymmetric slab.^[^ ^53^ ^]^ The tight‐binding calculations were based on the Su–Schrieffer–Heeger (SSH) model,^[^ ^54^, ^55^ ^]^ with parameters taken from ref [20]; a hopping integral of t_0_ = 0.4 eV, a spring constant of K = 0.55 eV/Å^2^, and an electron‐lattice coupling of α = 0.28 eV/Å.

## Conflict of Interest

The authors declare no conflict of interest.

## Author Contributions

T.I. performed STM experiments and analyzed the results. J.W.P. performed DFT and calculations. T.I. and H.W.Y. wrote the manuscript. H.W.Y. conceived the project idea, made the plan, and extracted the conclusions. T.I. and J.W.P contributed equally to the present work.

## Supporting information

Supporting Information

## Data Availability

The data that support the findings of this study are available from the corresponding author upon reasonable request.
