# Supplementary material for: Manipulating Individual Topological Solitons and Bisolitons in an Electronic System
Source: Adv Mater. 2025 Sep 25;38(3):e10318. doi: 10.1002/adma.202510318 (PMC12801370; doi:10.1002/adma.202510318)
Supplement: Supplementary file 1 — Supporting Information [file ADMA-38-e10318-s001.pdf]

ADVANCED MATERIALS

Supporting Information

for *Adv. Mater.*, DOI 10.1002/adma.202510318

Manipulating Individual Topological Solitons and Bisolitons in an Electronic System

*Taehwan Im, Jae Whan Park and Han Woong Yeom**

Supplementary information for "Manipulating individual topological solitons and bisolitons in an electronic system"

Tawhwan Im,[1,2] Jae Whan Park,[1] and Han Woong Yeom[1,2,*]

[1] *Center for Artificial Low Dimensional Electronic Systems, Institute for Basic Science (IBS), Pohang 37673, Republic of Korea*

[2] *Department of Physics, Pohang University of Science and Technology, Pohang 37673, Republic of Korea*

(Dated: August 27, 2025)

SUPPLEMENTARY NOTE 1 : TIGHT-BINDING MODEL

We investigated a tight-binding model of a double Peierls-dimerized atomic chain using the Su-Schrieffer-Heeger (SSH) Hamiltonian [1] for each chain and a zigzag interchain coupling term (Fig. S1(a)). This model was shown to describe well in both qualitative and quantitive levels the CDW state and solitons of In atomic wires on Si(111) [2]:

$$\hat{H} = \hat{H}^{(1)}_{\mathrm{SSH}} + \hat{H}^{(2)}_{\mathrm{SSH}} + \hat{H}_{\mathrm{coupling}} \tag{1}$$

Each SSH Hamiltonian consists of two components, the electron binding energy and interatomic lattice interactions:

$$\hat{H}^{(i)}_{\mathrm{SSH}} = \hat{H}^{(i)}_{\mathrm{el}} + \hat{H}^{(i)}_{\mathrm{latt}} \tag{2}$$

$$\hat{H}^{(i)}_{\mathrm{el}} = \sum_n t^{(i)}_{n,n+1} \left(c^{(i)\dagger}_n c^{(i)}_{n+1} + \mathrm{h.c.} \right) + \sum_n \varepsilon_n \, c^{(i)\dagger}_n c^{(i)}_n, \tag{3}$$

$$\hat{H}^{(i)}_{\mathrm{latt}} = \sum_n \frac{K}{2} \left(u_{n+1} - u_n \right)^2 + \sum_n \Gamma \left(u_{n+1} - u_n \right). \tag{4}$$

In the electronic part, t represents the nearest-neighbor hopping integral for electrons, $c^{(i)\dagger}_n$ and $c^{(i)}_n$ are the creation and annihilation operators, and ε_n denotes the site-dependent on-site energy. In the SSH model, the hopping integral depends linearly on the atomic distance as follows:

$$t = t_0 + \alpha \left(u_{n+1} - u_n \right), \tag{5}$$

where t_0 is the hopping integral of the undimerized configuration, u is the displacement of the n-th atomic site, and α is the electron-lattice coupling constant. In the lattice interaction, K is the spring constant described within the harmonic approximation, and Γ is an additional stretching force to the ends of the chain to prevent unexpected compensation effects [3–6]. The interchain hopping integrals were treated as a constant, determined by the parameter δ and assumed independent of the atomic site distance [2]:

$$\hat{H}_{\mathrm{coupling}} = \delta \sum_n \left(c^{(1)\dagger}_n c^{(2)}_n + c^{(2)\dagger}_n c^{(1)}_n \right). \tag{6}$$

The optimized parameters to describe the In atomic wires were adopted from Ref. [2]; a hopping integral of $t_0 = 0.4$ eV, a spring constant of $K = 0.55$ eV/Å^2, an electron-lattice coupling of $\alpha = 0.28$ eV/Å, interchain coupling of $\delta = 0.2$, and stretching force of $\Gamma = 0.39$ eV/Å. The model structure consists of 200 atomic sites per chain. To analyze structural properties of the CDW state shown in Fig. 1(b), all atoms were relaxed within the on-site energy of 21 sites, as illustrated in Fig. S1(b), until the residual force converged to within 0.01 eV/Å. The same model was used to get the soliton (domain wall) structures betwee four different CDW states as shown in Fig. S2.

SUPPLEMENTARY NOTE 2 : ESTIMATION OF ELECTRIC FIELD EFFECT

In the STM measurement, the tip is observed to lower by approximately 2 Å from the stable condition (-500 mV / 100 pA) to the soliton manipulation condition. However, directly measuring the absolute tip-sample distance remains challenging. To provide better understanding and enable direct comparison between experimental conditions and DFT calculations, we present a schematic showing the estimated current and electric field as a function of the tip-sample distance (Supplementary Fig. S8).

For the stable condition, we assume effective tip-sample distances of about 3–5 Å. At a shorter tip-sample distance of 3 Å (or 1 Å for the manipulation condition), the electric field and current are estimated to be approximately 0.2–0.3 eV/Å and 0.5 nA, respectively, at a bias voltage of -300 meV. Under these conditions, soliton manipulation is prevented due to the relatively small current-induced doping effect, and the CDW formation is stabilized by the electric field [Fig. 2(f)].

In contrast, at a bias voltage exceeding -400 meV, the CDW formation becomes destabilized as the electric field increases from 0.4 to 0.6 eV/Å. At -600 meV, the electric field reaches 0.6 eV/Å, leading to the complete disappearance of CDW stabilization and a reduction in the energy barrier due to the electric field (Supplementary Fig. S5). This theoretical interpretation, supported by DFT energetics and barrier calculations, aligns well with the experimental observations, where soliton manipulation requires a bias voltage exceeding -300 mV and matches the observed time scale.

For longer tip-sample distances of 4 and 5 Å [Supplementary Fig. S8(b) and 8(c)], the CDW formation under the electric field is most stabilized at -500 meV and -600 meV,

respectively. These findings are inconsistent with the experimental observations.

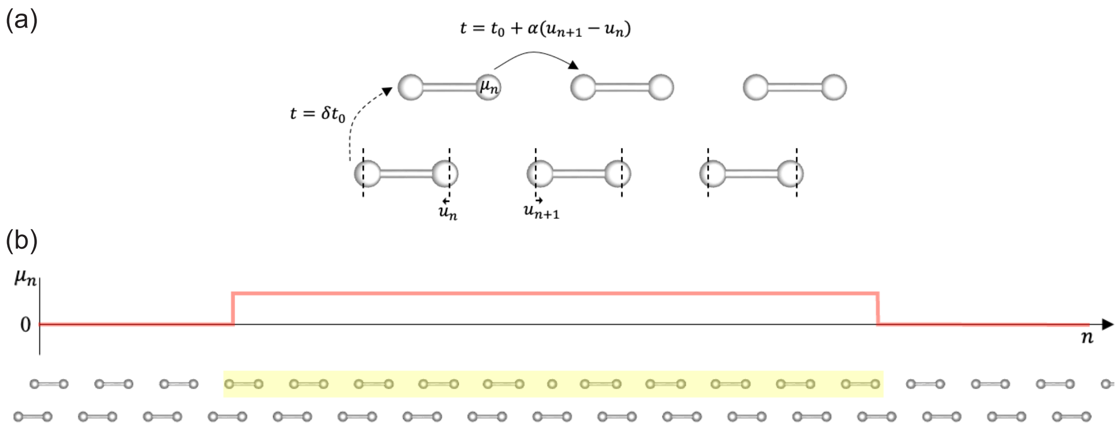

Fig. S1. **Schematic representation of the tight binding model for a double SSH chain.** (a) Schematic atomic structure of the chain. Vertical dashed lines indicate atomic positions in the undistorted configuration. (b) Illustration of the on-site energy tuning; the on-site energy of 21 atoms (highlighted in yellow) are adjusted uniformly, while those of the remaining atoms are set to zero.

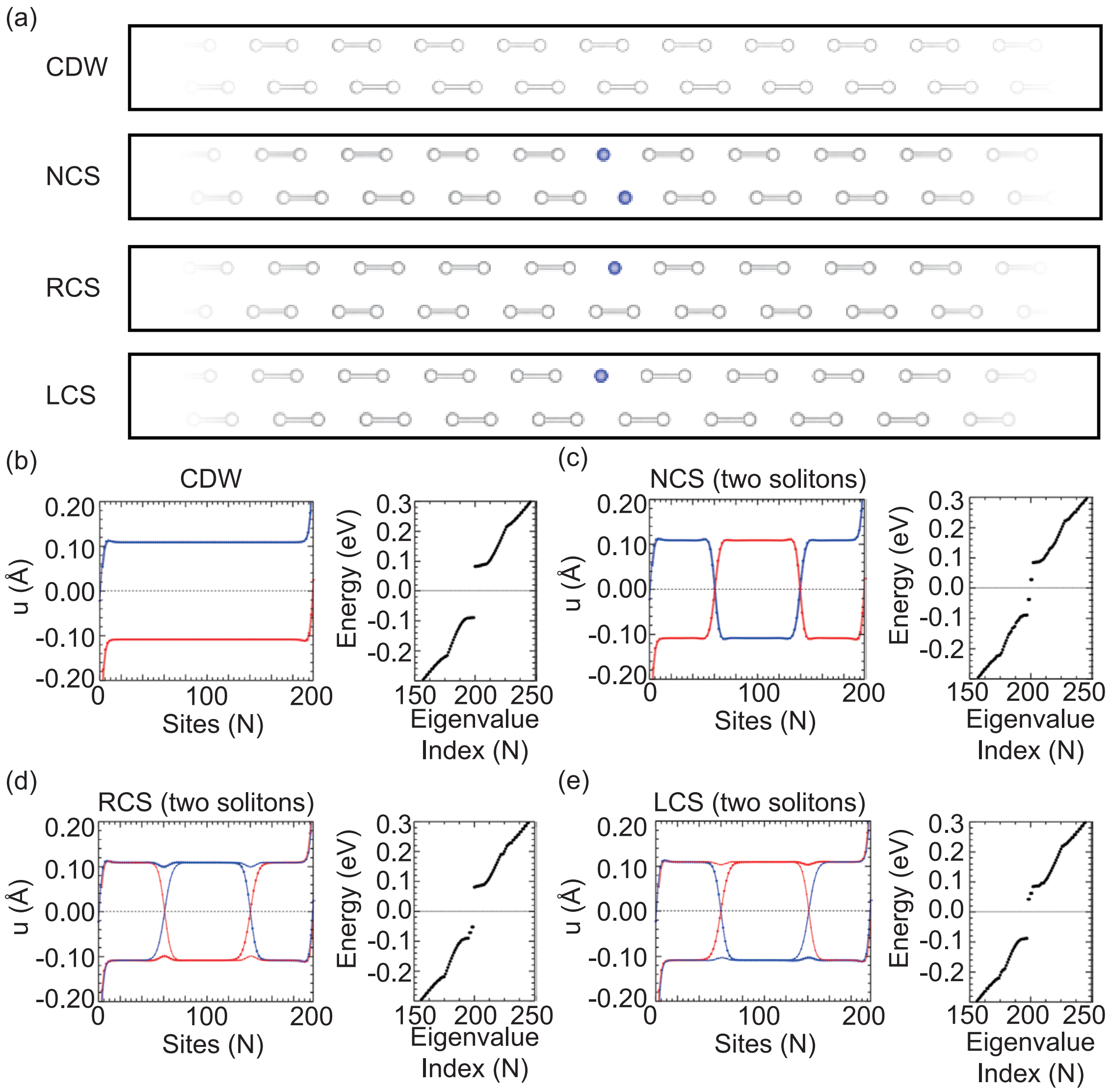

Fig. S2. **Tight-binding model for Z_4 solitons within a double SSH chain.** (a) Atomic structures of the bare CDW chain in one of the four degenerate CDW structures and those of non-chiral (NC), right-chiral (RC), and left-chiral (LC) solitons with the undimerized sites highlighted by blue balls. (b)-(e) Dimerization displacement profiles and energy eigenvalues corresponding to the CDW state (b), NC (c), RC (d), and LC (e) solitons. The dimerization displacement is defined as $u_n = x_n$ - $n \times a_0$, where n is atomic index, x is the atomic position and a_0 is the lattice constant. Each chain consists of 200 atoms for the CDW and NC soliton and 199 atoms for the RC and the LC solitons. To avoid unexpected edge states, the model is constructed with two identical solitons in each chain. The zero and the sign flip of the displacement indicate the soliton.

(a)

Z_4	0	-1	1	2
0	0	-1	1	2
-1	-1	2	0	1
1	1	0	2	-1
2	2	1	-1	0

(b)

+	G	L	R	N
G	G	L	R	N
L	L	N	G	R
R	R	G	N	L
N	N	R	L	G

Fig. S3. **Topological charges and algebraic relations of Z_4 solitons.** (a) Topological charge table for the Z_4 solitons in the indium atomic wire CDW system. The color-coded entries represent the topological charges associated with each soliton type: left-chiral (-1), right-chiral (1), non-chiral (2), and the ground state (0). (b) Addition table for Z_4 solitons showing the algebraic rules for combining different soliton types; left-chiral (L, red), right-chiral (R, blue), non-chiral (N, black), and the ground state (G, gray). The combination follows the Z_4 group structure, illustrating how solitons can transform into one another or annihilate depending on their topological charges. This figure are adapted from the Supplementary Information of Kim et al., Nature Physics 13, 444–447 (2017).

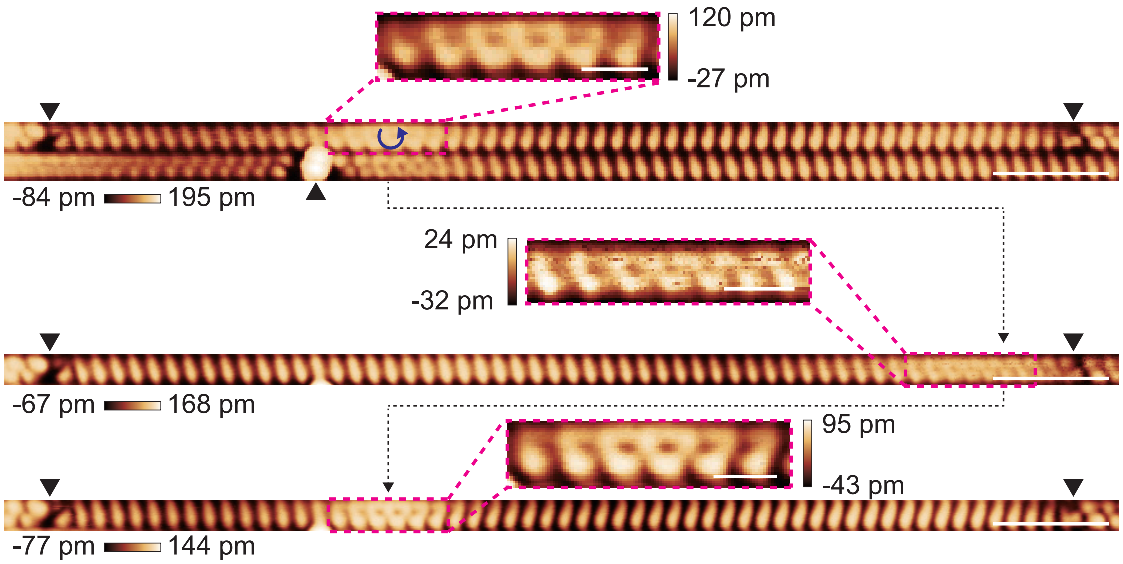

Fig. S4. **Translating a RC soliton from one pinning site to the other one by STM tunneling current.** 2D version of the STM images shown in Fig. 1d. Three defects (indicated by arrow heads) are shown in the two neighboring indium wires while two of them (one in the bottom wire and the other one in the right side of the top wire) are involved in trapping the soliton in the top wire. The soliton manipulated and tracked was marked by pink dashed boxes. This soliton corresponds to a right chiral (RC) soliton shown in Fig. 1c and its structure is consistently maintained for successive manipulations (see the enlarged images). These images were all taken at a moderate tunneling condition (tunneling bias -500 mV and tunneling current 100 pA) and at 78 K. Between these three images, the tip was approached on top of the soliton to access the tunneling condition for manipulation (-500 mV bias with 0.9 nA) and scanned repeatedly over the soliton along a line of a narrowed width of 2 nm until the soliton moves. Such repeated line scan data are shown in Fig. S4. The scale bars represent 5 nm, while those in the enlarged images represent 1 nm.

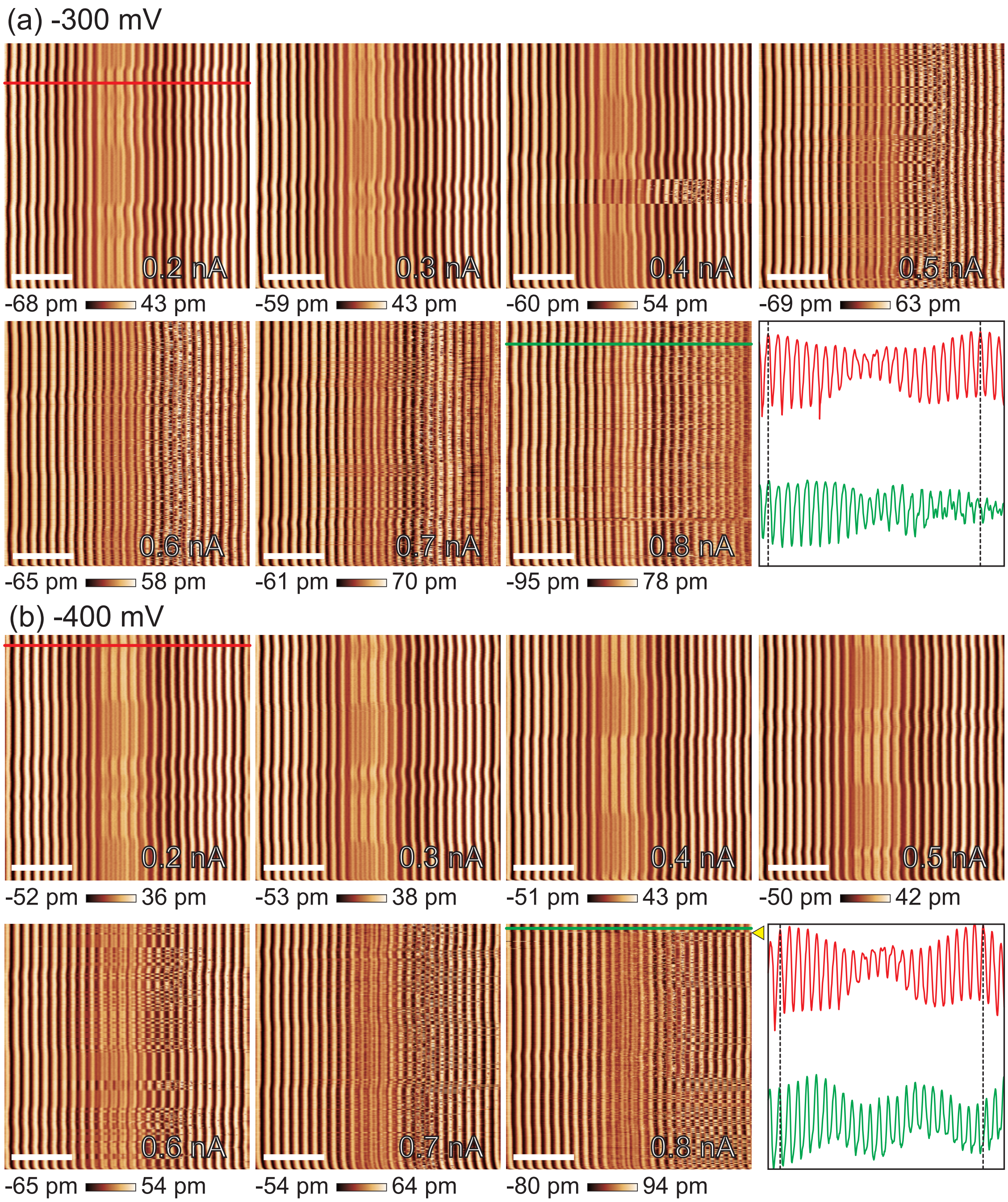

(a) -300 mV
0.2 nA
0.3 nA
0.4 nA
0.5 nA
-68 pm 43 pm
-59 pm 43 pm
-60 pm 54 pm
-69 pm 63 pm
0.6 nA
0.7 nA
0.8 nA
-65 pm 58 pm
-61 pm 70 pm
-95 pm 78 pm
(b) -400 mV
0.2 nA
0.3 nA
0.4 nA
0.5 nA
-52 pm 36 pm
-53 pm 38 pm
-51 pm 43 pm
-50 pm 42 pm
0.6 nA
0.7 nA
0.8 nA
-65 pm 54 pm
-54 pm 64 pm
-80 pm 94 pm

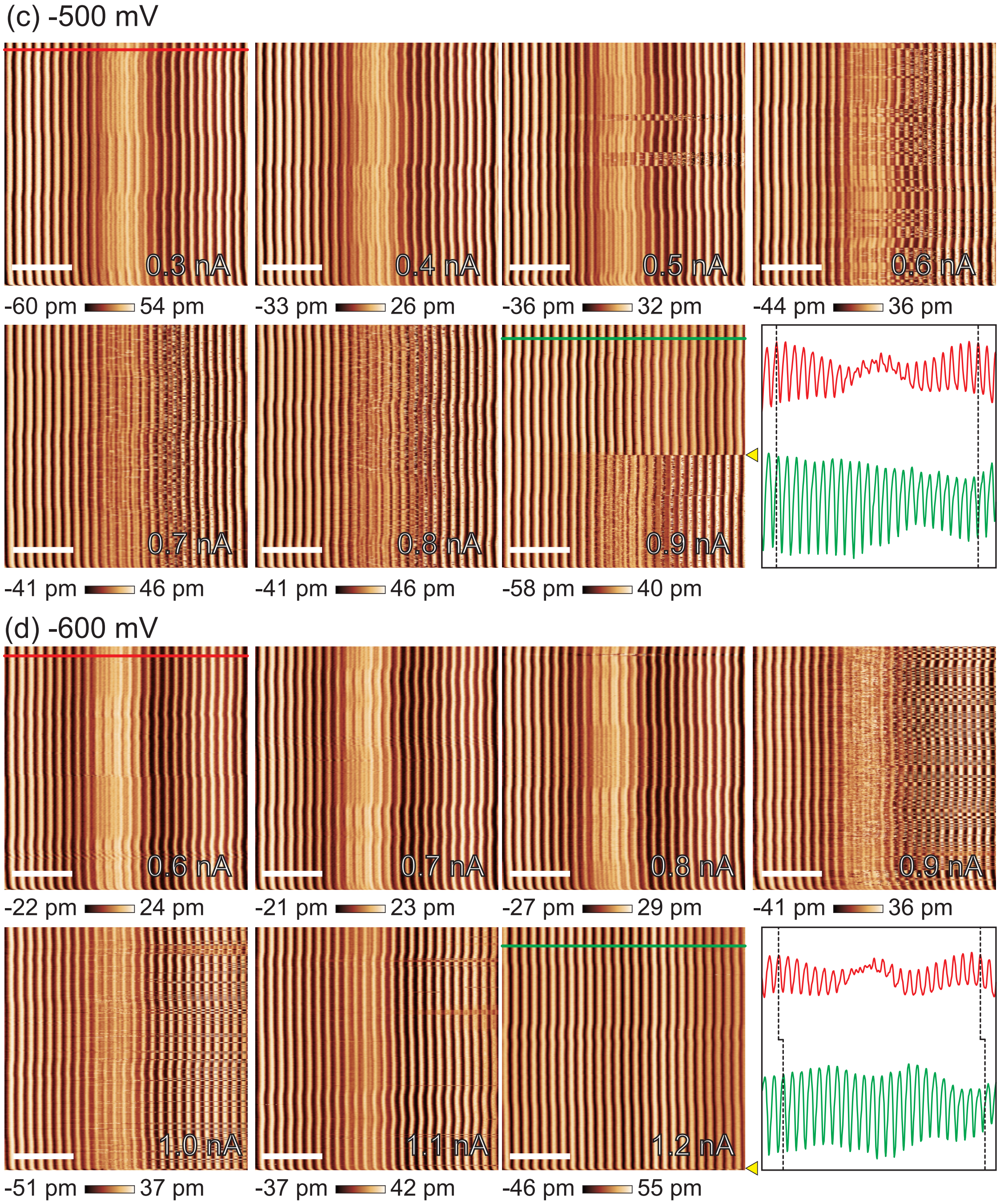
(c) -500 mV
0.3 nA
0.4 nA
0.5 nA
0.6 nA
-60 pm
54 pm
-33 pm
26 pm
-36 pm
32 pm
-44 pm
36 pm
0.7 nA
0.8 nA
0.9 nA
-41 pm
46 pm
-41 pm
46 pm
-58 pm
40 pm
(d) -600 mV
0.6 nA
0.7 nA
0.8 nA
0.9 nA
-22 pm
24 pm
-21 pm
23 pm
-27 pm
29 pm
-41 pm
36 pm
1.0 nA
1.1 nA
1.2 nA
-51 pm
37 pm
-37 pm
42 pm
-46 pm
55 pm

Fig. S5. **Investigation of STM manipulation conditions over a soliton.** To explore the manipulation conditions, we repeated STM line scan for a segment of the indium wire (20 nm in length) with a soliton in its center. We, then, increased the tunneling current stepwise by 100 pA at a fixed tunneling bias of (a) -300 mV, (b) -400 mV, (c) -500 mV, and (d) -600 mV until the soliton moves. The images are the STM line scans (the horizontal axis) stacked in time (the vertical axis) for given tunneling bias and current. The initial soliton position can be seen in a single STM line profile (red curves cut along the red line in the first image of each figure). In the time-stacked images, the soliton position is represented by the blurry stripes of a different contrast in the center of the images. Under -300 mV bias in (a), the soliton did not move up to the current of 0.8 nA (or higher, not shown here) as confirmed by the green line profile. As increasing the tunneling current, the CDW strucutre fluctuates dynamically as shown in the images and in the green line profiles. For a higher bias, the soliton moves at a high current condition (for example, 0.8 nA for -400 mV in (b)). The moment of the soliton motion (moving out of this line profiled segment) was marked by yellow arrow head. The absence of the soliton can be seen clearly in the green line profile taken after the soliton motion. By comparing the red and green line profiles before and after the soliton motion, one can confirm the disappearance of the CDW phase shift (as guided by the vertical dashed lines), the footprint of a soliton. The white scale bar represents 5 nm.

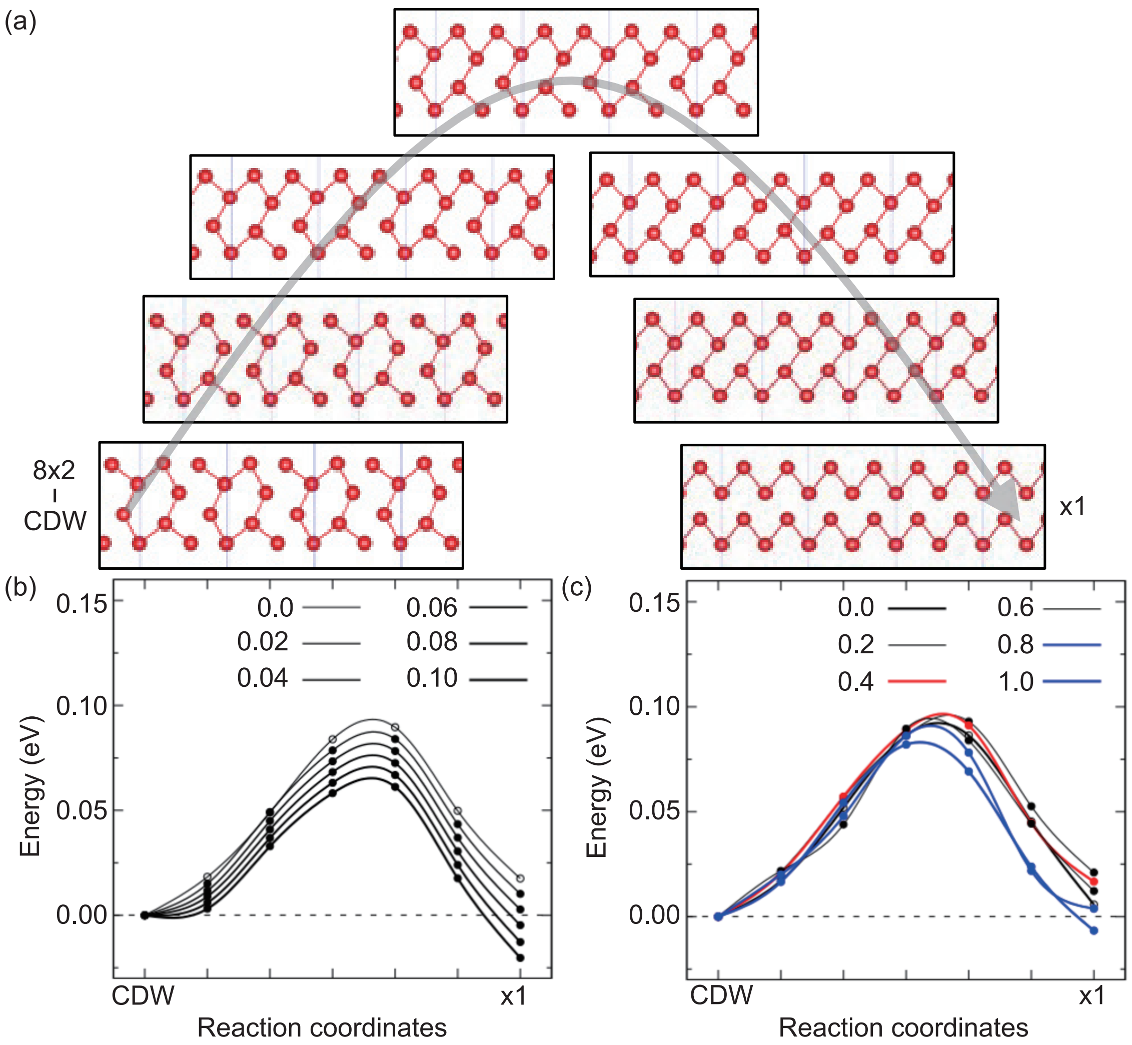

Fig. S6. **Energy barriers between the insulating 4x2 CDW phase to the metallic 4x1 phase in DFT calculations.** (a) Atomic structures illustrating the phase transition from the 4x2 CDW to the 4x1 metallic phase, featuring five intermediate atomic configurations obtained through linear interpolation. (b) The total energies for the atomic configurations given in (a) calculated for different amounts of hole doping. These calculations indicates the existence of the energy barrier for the transition between the the 4x2 CDW to the 4x1 metallic phase, which decreases monotonically as the hope doping increases. (c) The transition energy barrier as a function of electric field. The energy barrier increases for low field but decreases a a higher field beyond 0.06 e/Å. This trend is consistent with the energy variations shown in Fig. 2(f).

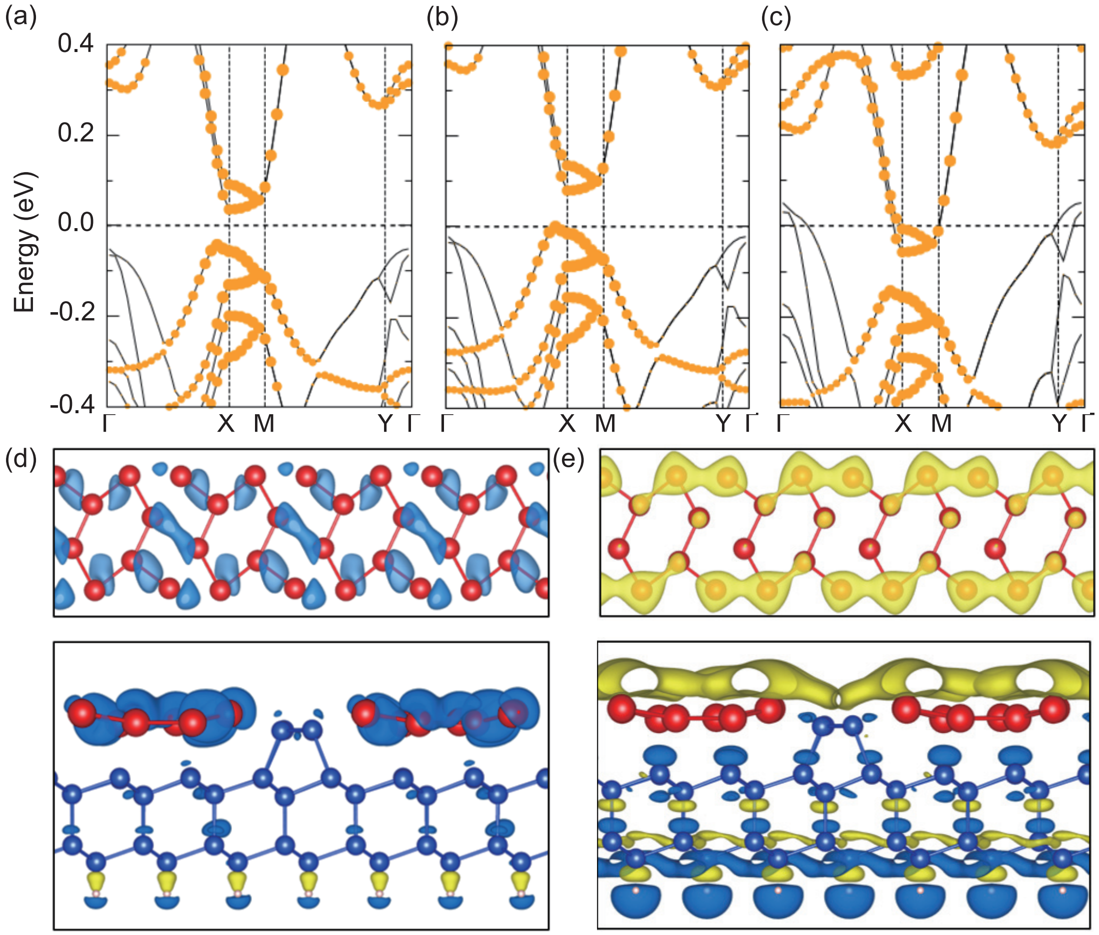

Fig. S7. **Comparison of the effects of hole doping and electric field in DFT calculations.** (a)-(c) DFT band structures of the pristine 8x2-CDW surface (a), with hole doping of 0.06/cell (b), and with an electric-field of 1.0 eV/Å (c). The size of symbols is proportional to the weight of the corresponding electronic wave function localized at In atoms. (d), (e) Total charge difference form the pristine surface for (d) the hole doping of 0.06/cell and (e) the electric field of 1.0 eV/Å. Top and bottom panels represent top and side views, respectively. Yellow and blue isosurfaces indicate the positive and negative charge difference, respectively. The isosurface value is 2×10^{-5} e/bohr^3 for (d) and 5×10^{-4} and 3×10^{-4} e/bohr^3 for the top and side views in (e), respectively.

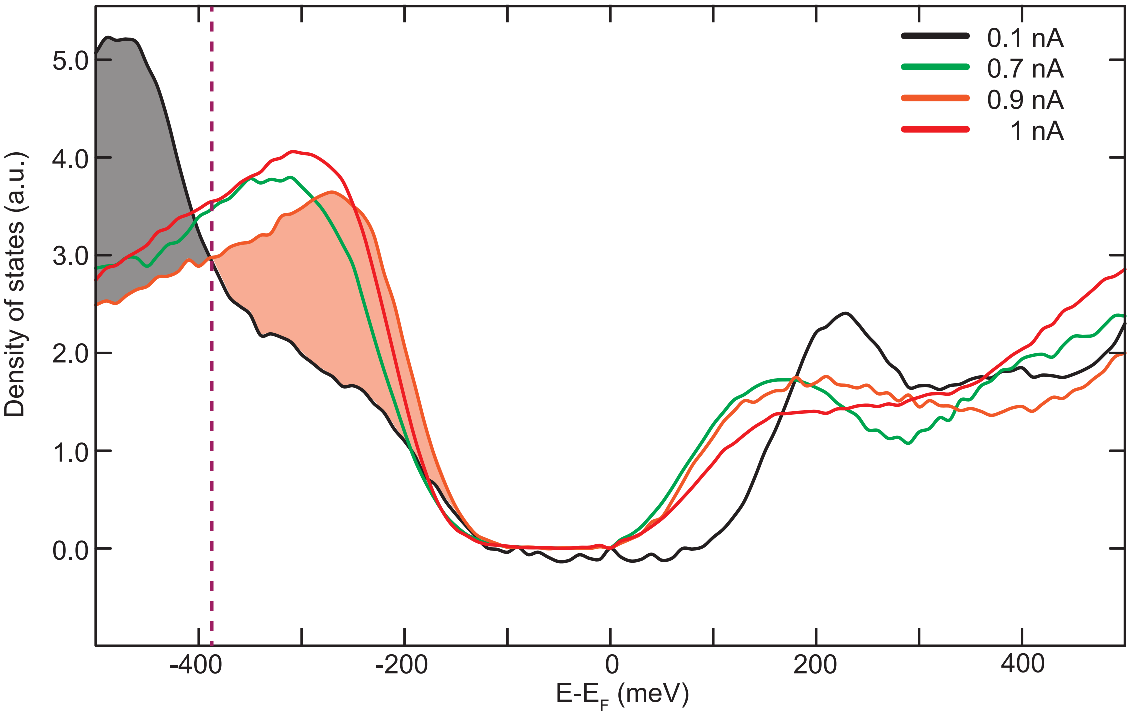

Fig. S8. **Changes of electronic states of the indium wire at different tunneling currents.** Comparison of the tunneling spectroscopy data measured at tunneling currents of 100 pA (black), 700 pA (green), 900 pA (orange), and 1000 pA (red). The band gap was reduced significantly for the high tunneling current, which indicates that the CDW order is suppressed. For the low (high) negative bias below (above) -400 mV, the electron density of states is increased (decreases) which would reduce (enhances) the transient hole doping effect.

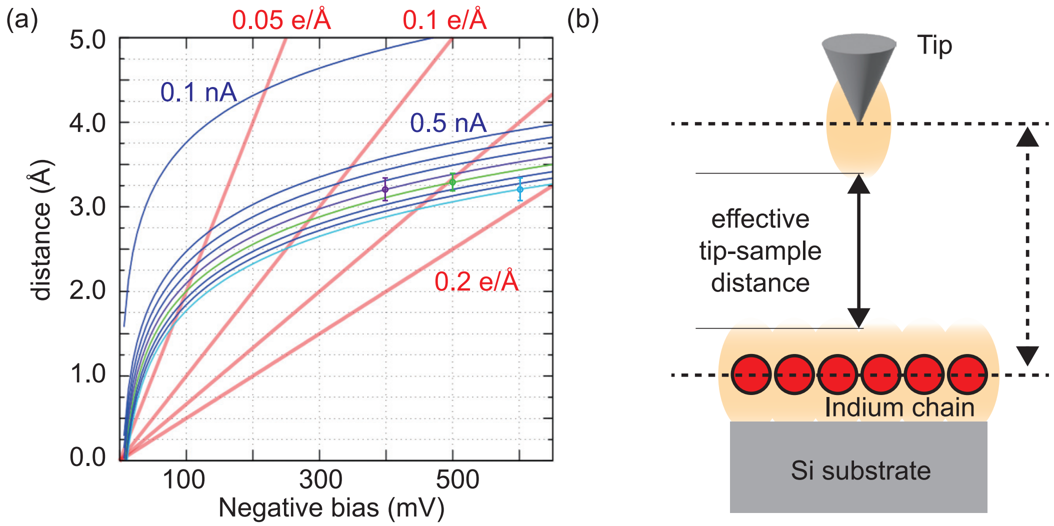

Fig. S9. **Estimation of the tunneling current and the electric field for different tip-sample distances and bias voltages.** The effective tip-sample distance (the distance between the tunneling electron clouds of the tip and the sample shown in (b)) is 5 Å (a), which are estimated at the topographic imaging condition of bias voltage -500 mV and tunneling current 0.1 nA. The blue curves show how the tip-sample distance changes for different bias and tunneling currents (0.1, 0.5. 0.6, 0.7, 0.8, 0.9, 1.0, 1.1, and 1.2 nA). We used a simple formula $I = c_1 V e^{-c_2 d}$ where, $c_1 = 1.25$ and $c_2 = 7$, V is the bias voltage. c_1 and c_2 are determined to fit the above distance estimation. One can see that the distance changes by approximately 2 Å from 0.1 nA to 0.8-1.2 nA for the bias range of 400-600 mV, which agrees well with the experimental situation. The red lines indicate the electric-field strength ($E = V/d$), which changes from about 0.15 e/Å to 0.6 e/Å at maximum. (b) Schematic representation of the effective tip-sample distance. Dashed (solid) arrows indicate actual tip-sample distance, while solid arrows represent the effective distance between the edge of electron clouds (yellow shadings).

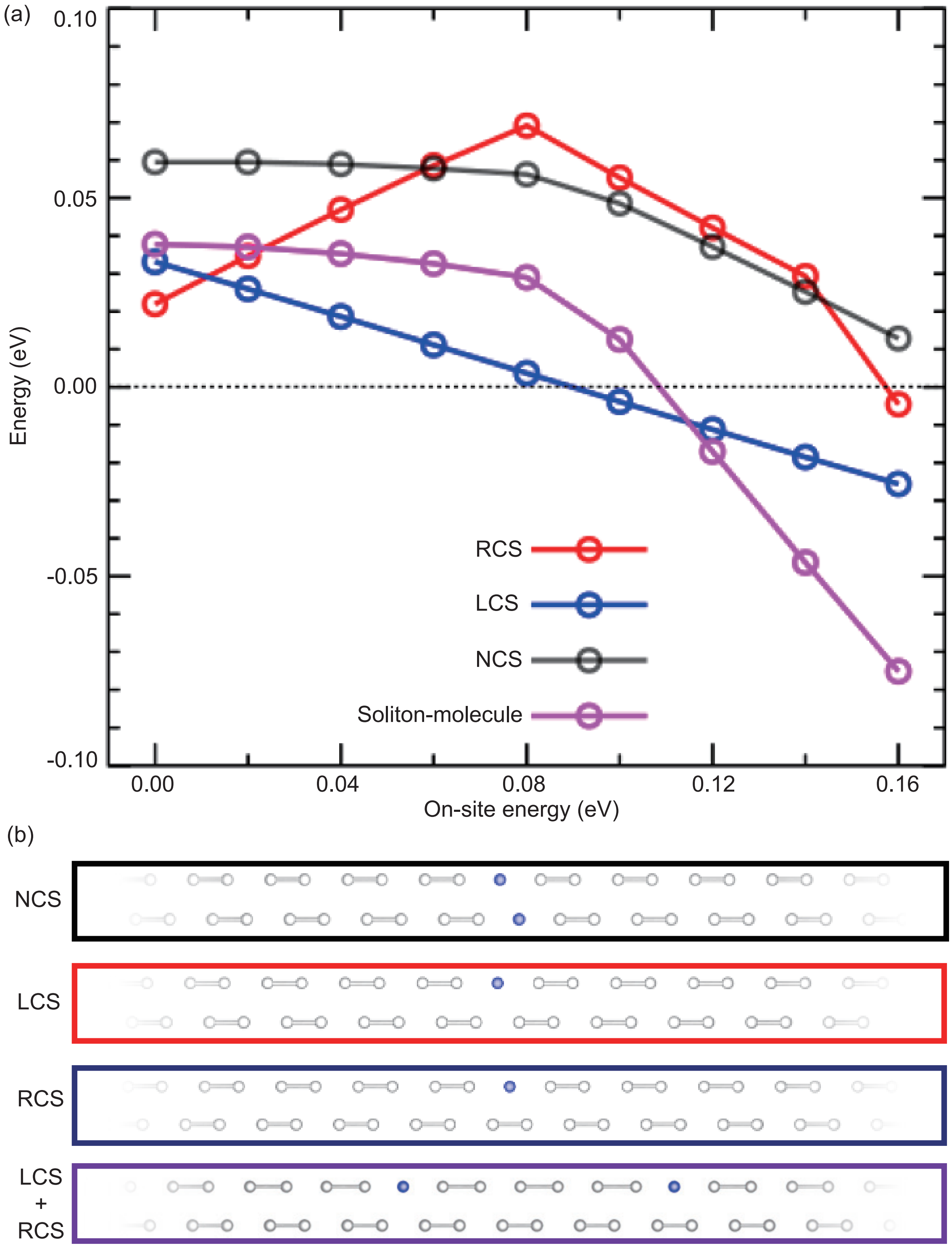
(a)
0.10
0.05
0.00
-0.05
-0.10
Energy (eV)
0.00
0.04
0.08
0.12
0.16
On-site energy (eV)
RCS
LCS
NCS
Soliton-molecule
(b)
NCS
LCS
RCS
LCS
+
RCS

Fig. S10. (a) Tight-binding energetics for RC, LC, NC solitons and a soliton molecule as function of on-site energy (see Fig. S1). (b) Atomic structures of AC, RC, and LC solitons, as well as a RC + LC soliton (see Fig. S10). All atomic structures are fixed at their isolated equilibrium positions (see Fig. S2). For the soliton molecule, the structure is fixed at the fully-relaxed configuration obtained with an on-site energy of 0.16 eV at which a soliton molecule is formed (see Fig. 3 in the main text and Fig. S10).

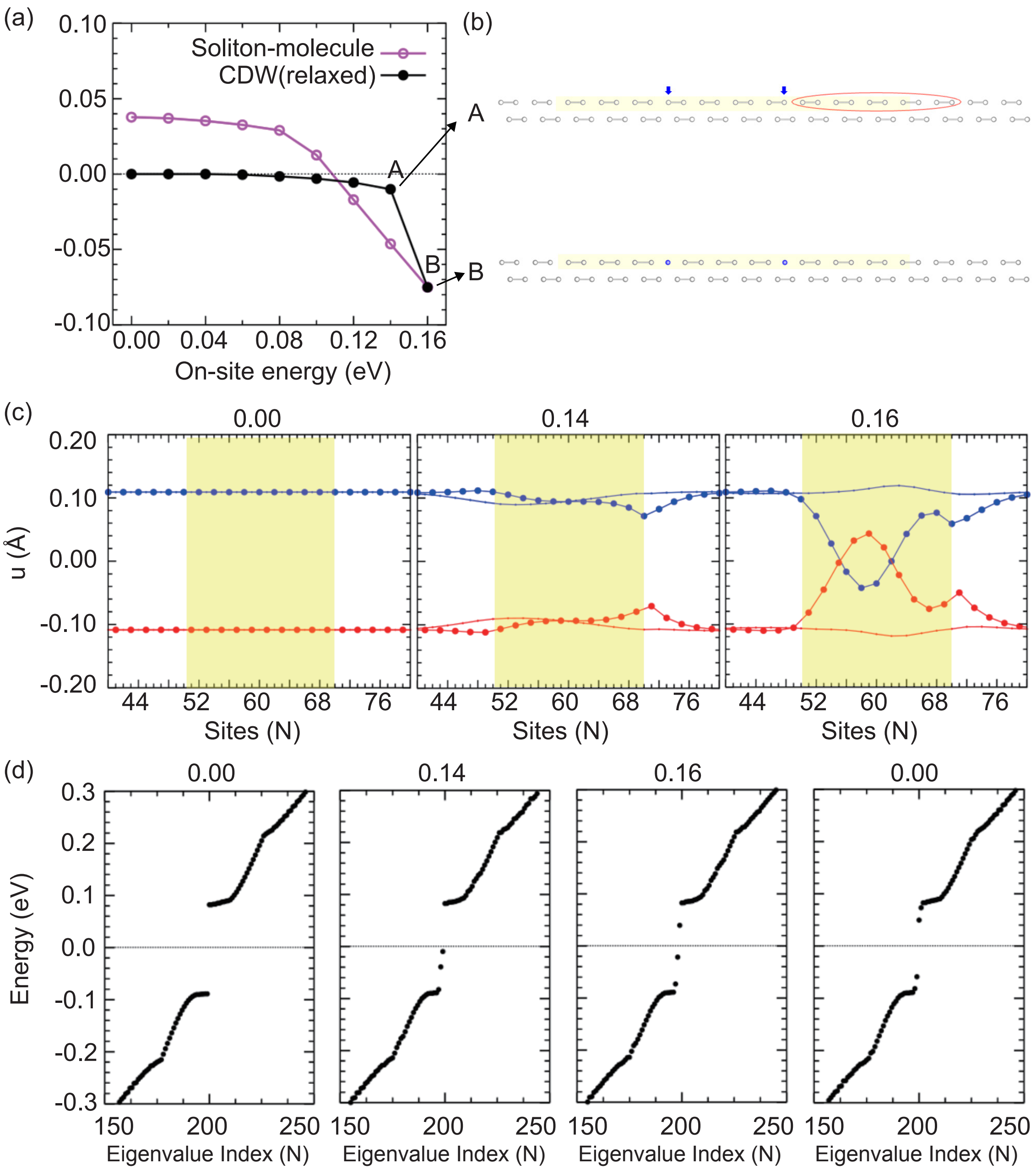

(a)
(b)
Soliton-molecule
CDW(relaxed)
A
B
On-site energy (eV)
(c)
0.00
0.14
0.16
u (Å)
Sites (N)
(d)
Energy (eV)
Eigenvalue Index (N)

Fig. S11. **Formation of a soliton molecule in the tight-binding model.** (a) Energetics of a soliton molecule as function of on-site energy, similar to Fig. 2(e). (b) Atomic structures of the double SSH chain (Fig. S1) at an on-site energy of 0.14 (A) and 0.16 eV (B). Yellow shading highlights the region where the local on-site energy changes. At 0.14 eV, the CDW structure is preserved, except for a slight reduction in the CDW amplitude in the boundary region on the right side (highlighted by a red oval; see also (c)). At 0.16 eV, a RC and a LC soliton are formed with their positions highlighted by blue circles. For comparison, the same sites are indicated by blue arrows in the 0.14 eV structure. (c) Atomic displacement profiles for given on-site energies of 0, 0.14, and 0.16 eV. The bifurcated values indicate the dimerization along the chains. Large and small circles represent one of indium chains of the double indium chain. Yellow shading highlights the region where the local on-site energy changes. The reduced dimerization is shown for 0.14 eV but the two zero displacement sites on one chain are found at 0.16 eV, which correspond to solitons shown in (b). (d) Energy eigenvalues obtained in the tight binding model for different on-site energies. The last panel corresponds to the soliton-molecule states, showing the atomic structure obtained from the 0.16 eV case but with the perturbed on-site energy removed. Two in-gap states in both empty and filled states indicate the formation of soliton molecular states.

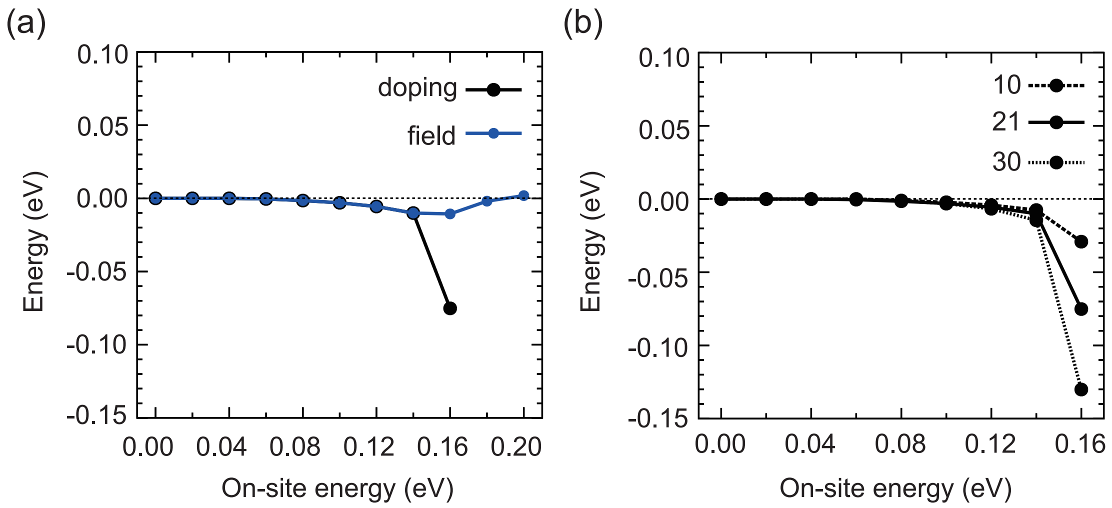

Fig. S12. **Effect of the change in on-site energy within the tight-binding model.** (a) Variation of the total energy under doping and electric field on the pristine CDW structure through the change in on-site energy. For the doping effect, once the Fermi level is fixed, the band filling changes according to the on-site energy, which corresponds to doping. The electric field effect ($\pm$V) can be expressed in terms of the chemical potential as $= \pm$eV [Physical Review **B** 111, 045405 (2025)]. To isolate the pure electric field effect, we maintained an overall half-filled condition so that no doping occurs. Doping induces soliton pair formation at an on-site energy of 0.16 eV, whereas the CDW ground state is preserved under the field effect. (b) As mentioned in Fig. S11, the on-site energy change was introduced locally for 21 sites. We checked how the the effect of varying the number of perturbed sites to 10 (3.8 nm) and 30 (11.4 nm). In all three cases, the solitons were created at 0.16 eV, but the energy change decreases with increasing perturbation length, consistent with the strong localization of solitons. This indicates that our results obtained by the on-site energy change are robust.

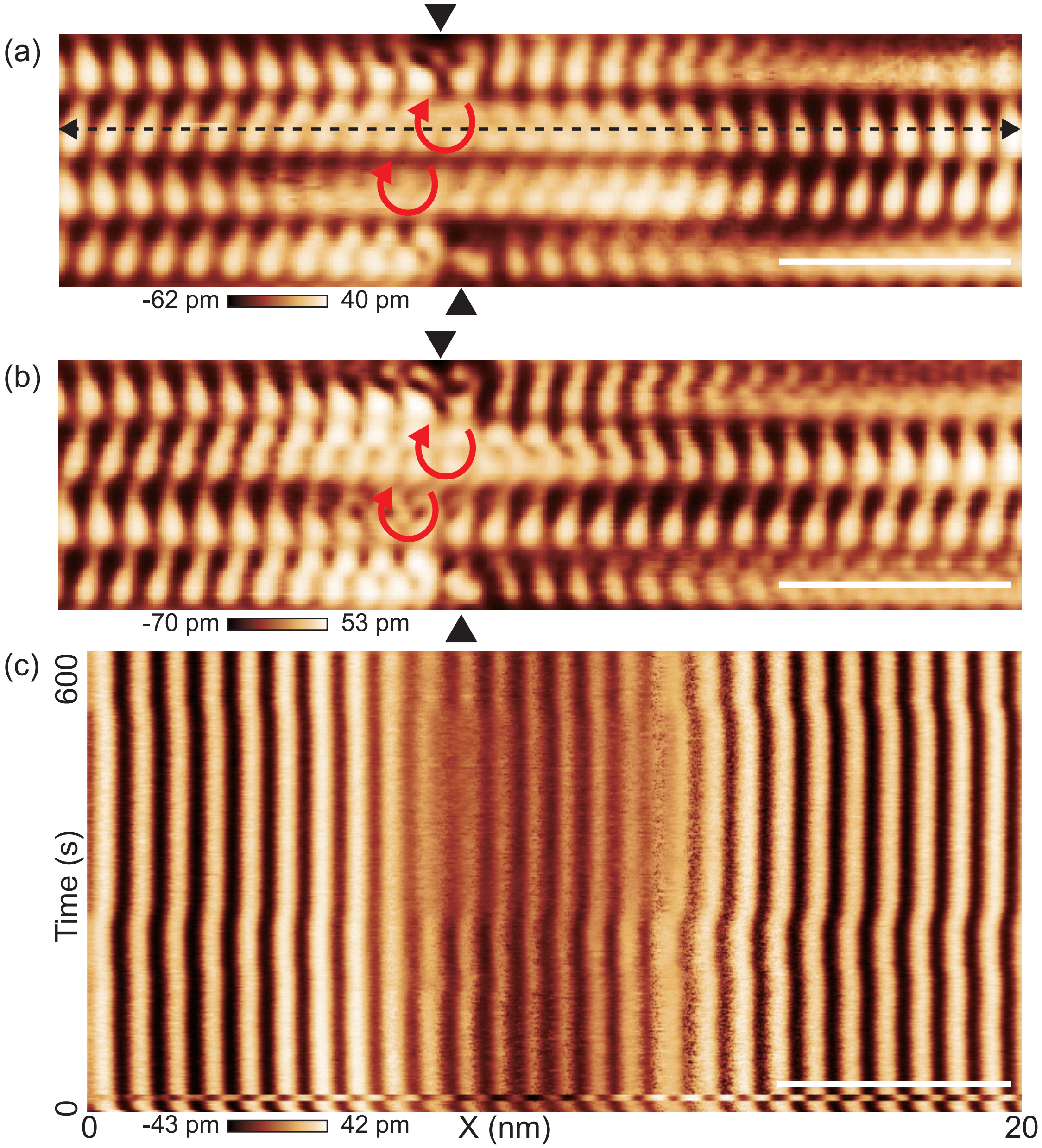

Fig. S13. **The challenge of manipulating LC solitons pinned by an interwire defect.** (a) STM image measured at -500 mV / 100 pA, showing two LC solitons pinned by interwire defects indicated by arrow heads. (b) STM image measured at -500 mV / 1 nA, which is typically used for the manipulation of an RC soliton. (c) Sequential line scan image measured at -500 mV / 1 nA along the black arrow in (a). Although a brief sign of fluctuation was observed at the beginning of the scan, the soliton structure was found to remain highly stable throughout. All STM images were captured at 78 K and the scale bars represent 5 nm.

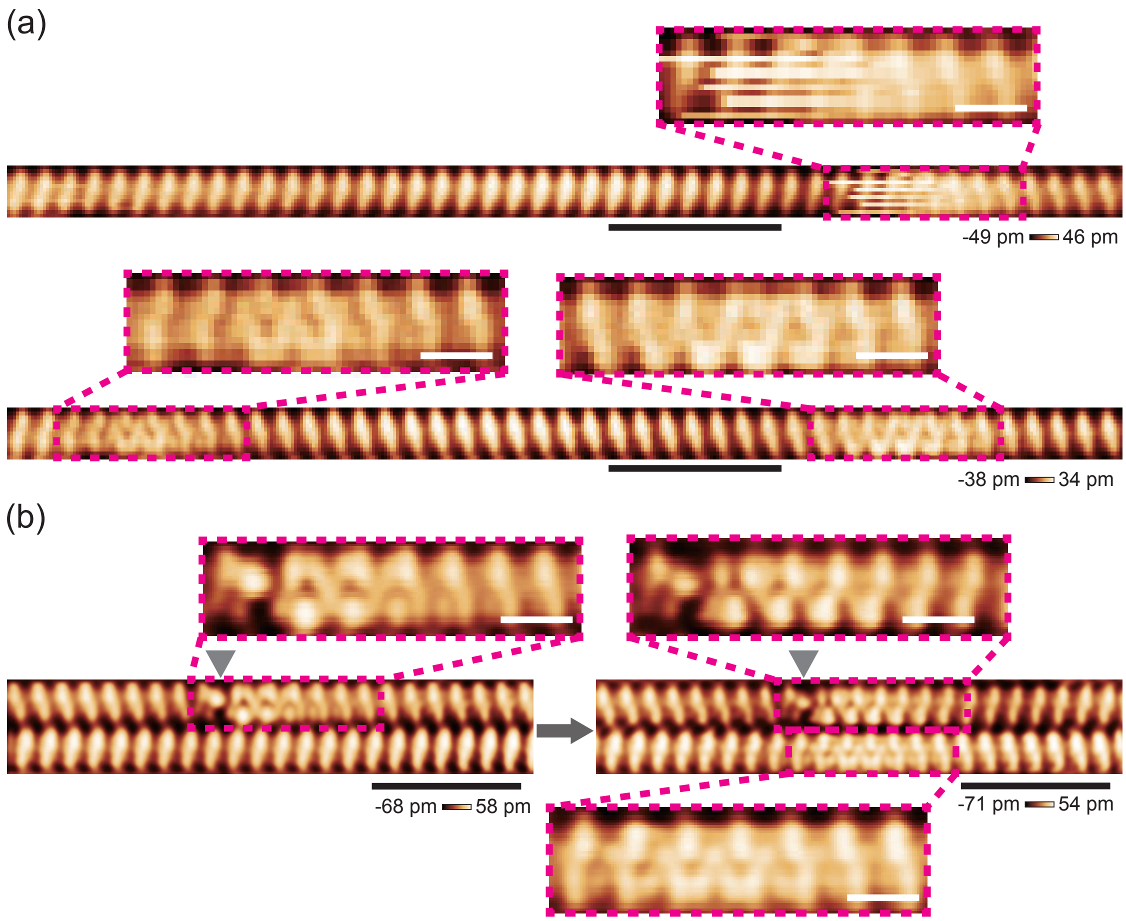
(a)
-49 pm
46 pm
-38 pm
34 pm
(b)
-68 pm
58 pm
-71 pm
54 pm

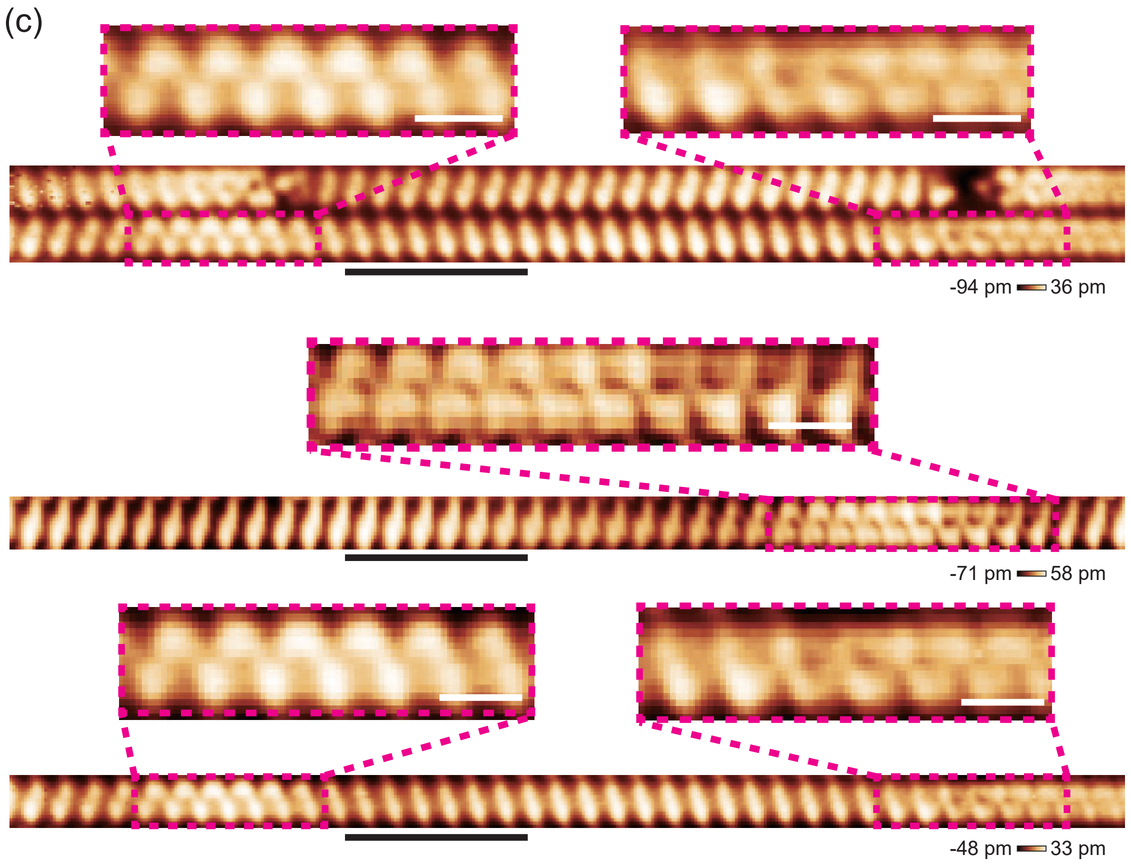

Fig. S14. **Enlarged STM images corresponding to the manipulation processes shown in Fig. 4 of the main text.** (a–c) Magnified views of the soliton manipulations displayed in Fig. 4(a–c), respectively, provided to clearly resolve the soliton structures and their transformations. The white scale bars in the enlarged images correspond to 1 nm, while the black scale bars in the original STM images correspond to 5 nm. Each magnified image corresponds to the region indicated by the pink dashed boxes in the STM images.

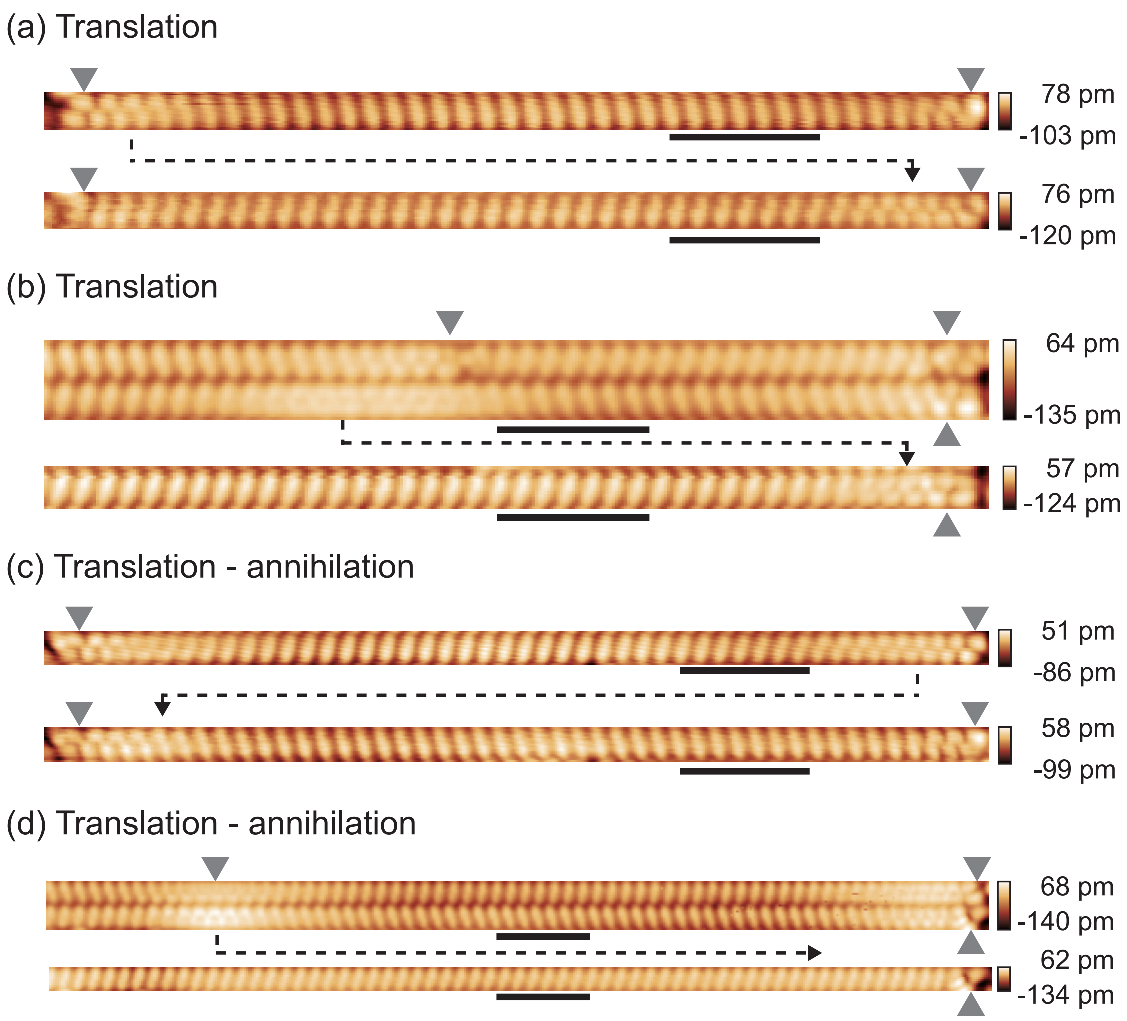
(a) Translation
78 pm
-103 pm
76 pm
-120 pm
(b) Translation
64 pm
-135 pm
57 pm
-124 pm
(c) Translation - annihilation
51 pm
-86 pm
58 pm
-99 pm
(d) Translation - annihilation
68 pm
-140 pm
62 pm
-134 pm

(e) Translation - Molecule

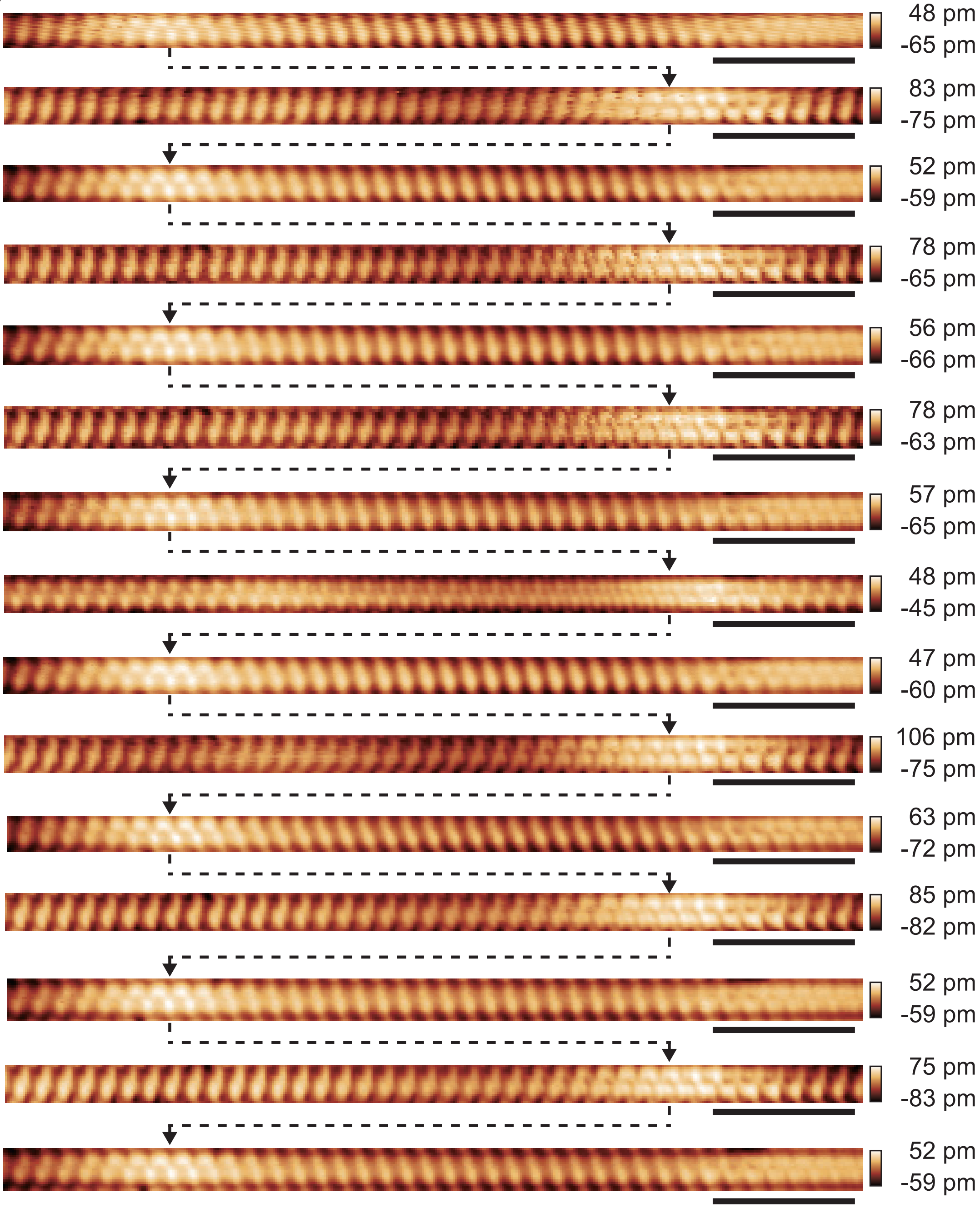

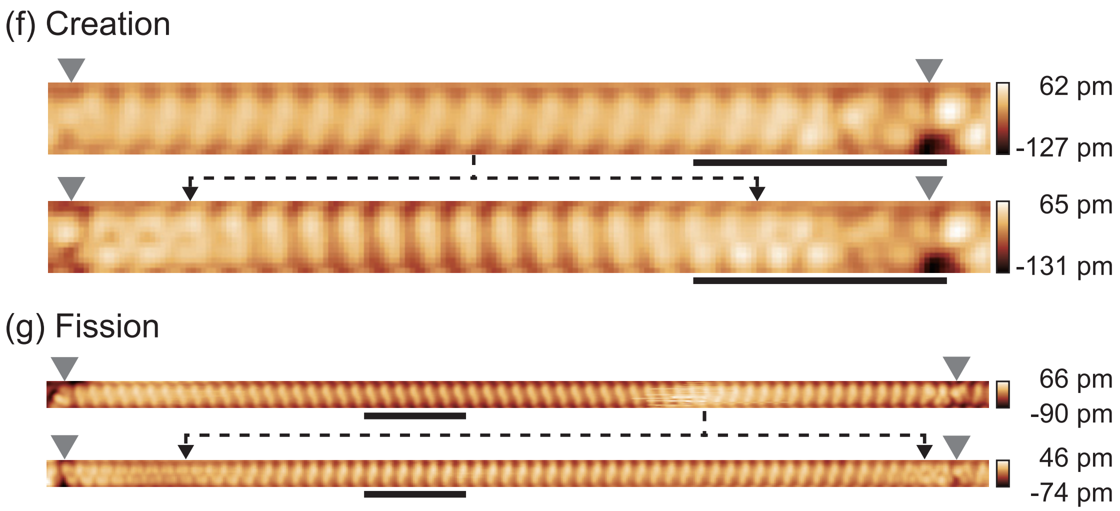

Fig. S15. **Representative STM images showing various soliton manipulations under identical conditions.** (a) Translation of a soliton located at a wire termination to the opposite end of the wire. (b) Depinning of a soliton trapped initially by an intrawire defect and its subsequent translation to the wire end. (c) Translation of a soliton at a wire termination to an opposite-chirality soliton at the other end of the wire leads to pair annihilation. (d) Depinning of a soliton pinned by an interwire defect, followed by its translation to the wire end and annihilation with a soliton at a wire termination. (e) Repeated depinning and translation of a RC soliton to a nearby LC soliton site (which is pinned by a defect in the upper wire not shown here) to form a soliton molecule and reversing the process (dissociating the RC soliton and translating back to its original pinning site). (f) Creation of an RC–LC soliton pair from a pristine CDW segment under hole injection. (g) Fission of a fluctuating RC soliton (noted by the noisy STM image) into LC and NC solitons. All STM images were taken at 78 K, and the scale bars represent 5 nm. Black dashed arrows indicate soliton motion and transformation, while gray triangles denote defects. All manipulations were performed under the tunneling conditions described in the main text.

* yeom@postech.ac.kr

[1] W.-P. Su, J. Schrieffer, and A. Heeger, Soliton excitations in polyacetylene, Physical Review B **22**, 2099 (1980).

[2] S. Cheon, T.-H. Kim, S.-H. Lee, and H. W. Yeom, Chiral solitons in a coupled double peierls chain, Science **350**, 182 (2015).

[3] W. Su and J. Schrieffer, Soliton dynamics in polyacetylene, Proceedings of the National Academy of Sciences **77**, 5626 (1980).

[4] D. Vanderbilt and E. J. Mele, Effects of disorder on the electronic structure of undoped polyacetylene, Physical Review B **22**, 3939 (1980).

[5] F. L. Vos, D. P. Aalberts, and W. van Saarloos, Su-schrieffer-heeger model applied to chains of finite length, Physical Review B **53**, 14922 (1996).

[6] L. M. Arancibia, A. I. Bertoni, C. G. Sánchez, and A. M. Lobos, Towards electrical domain-wall control in polyacetylene-based electronic nanodevices, arXiv preprint arXiv:2404.15257 (2024).
